# *In Vivo* Tau Imaging for a Diagnostic Platform of Tauopathy Using the rTg4510 Mouse Line

**DOI:** 10.3389/fneur.2017.00663

**Published:** 2017-12-07

**Authors:** Naruhiko Sahara, Masafumi Shimojo, Maiko Ono, Hiroyuki Takuwa, Marcelo Febo, Makoto Higuchi, Tetsuya Suhara

**Affiliations:** ^1^Department of Functional Brain Imaging Research, National Institute of Radiological Sciences, National Institutes for Quantum and Radiological Science and Technology, Chiba, Japan; ^2^Department of Psychiatry and Neuroscience, University of Florida College of Medicine, Gainesville, FL, United States

**Keywords:** tau protein, transgenic mouse, positron emission tomography, magnetic resonance imaging, two-photon microscopy

## Abstract

Association of tau deposition with neurodegeneration in Alzheimer’s disease (AD) and related tau-positive neurological disorders collectively referred to as tauopathies indicates contribution of tau aggregates to neurotoxicity. The discovery of tau gene mutations in FTDP-17-*tau* kindreds has provided unequivocal evidence that tau abnormalities alone can induce neurodegenerative disorders. Therefore, visualization of tau accumulation would offer a reliable, objective index to aid in the diagnosis of tauopathy and to assess the disease progression. Positron emission tomography (PET) imaging of tau lesions is currently available using several tau PET ligands. Because most tau PET ligands have the property of an extrinsic fluorescent dye, these ligands are considered to be useful for both PET and fluorescence imaging. In addition, small-animal magnetic resonance imaging (MRI) is available for both structural and functional imaging. Using these advanced imaging techniques, *in vivo* studies on a mouse model of tauopathy will provide significant insight into the translational research of neurodegenerative diseases. In this review, we will discuss the utilities of PET, MRI, and fluorescence imaging for evaluating the disease progression of tauopathy.

## Introduction

Dementia is a leading cause of death in developed countries. Increasing age is the greatest risk factor for dementia. About 46 million people in the world are estimated to be suffering from dementia, and this figure is expected to rise to 135 million by 2050. Alzheimer’s disease (AD) is the most common type of dementia. The global impact of AD will strikingly affect social and economic costs. Distinctive features of AD are the deposited accumulations of β-amyloid (Aβ) protein fragments and intracellular neurofibrillary tangles (NFTs) (1). NTFs are closely associated with the severity of brain function loss in AD (2). Therefore, making tau protein a target in the treatment of AD has become a major therapeutic strategy.

Recent advances in positron emission tomography (PET) imaging research have led to significant breakthroughs with the application of newly developed tracers for visualizing regional tau depositions (3–8). This technology allows us to non-invasively evaluate the progression of tau pathology in living brains. PBB3 was developed as a novel PET tracer that binds with tau for the diagnosis of AD and other neurodegenerative diseases regarded as tauopathies (4, 9). PET imaging using [^11^C]PBB3 (radiotracer) is superior in detecting tau deposits, and it is closely aligned with disease symptoms. This was also confirmed by our tauopathy mouse models (4; Ishikawa, forthcoming^1^). ^11^C-labeled tracers, due to their short half-life, are generally unsuitable for clinical practice, but [^11^C]PBB3 can be used to evaluate certain tau conditions including AD (4, 10). The fluorescent quality of PBB3 makes it especially useful for multimodal imaging, allowing us to assess its binding by microscopic observation of related cells and tissues.

Animal models of tauopathy are essential for preclinical studies of AD and related dementias. The rTg4510 mouse line was developed to model aspects of tauopathy through forebrain expression of the P301L mutated human tau (11). Expression of human tau is controlled by the tetracycline transactivator transgene under the CaMKIIα promoter. This mouse line develops progressive intracellular tau aggregations in corticolimbic areas and forebrain atrophy (11). The age-dependent tau pathology of rTg4510 mice has been investigated in detail by immunohistochemical and biochemical examinations (12–14). These postmortem brain-based studies showed an extensive increase of pathological tau inclusions in the cerebral cortex and hippocampus between 4 and 6 months of age. Now, for both current and therapeutic strategies, rather than endpoint measurements, researchers are eagerly involved in the development of new *in vivo* protocols for tracking the progressive pathological status in living animals.

Taking advantage of the multimodality of the PBB3 ligand, *in vivo* monitoring of NFT formation is now available for possible in tauopathy mouse models, including the rTg4510 mouse. In addition, advanced brain magnetic resonance imaging (MRI) techniques have enabled us to visualize neuronal dysfunction and the structural changes related to neurodegenerative processes in living animals. Here, we will introduce *in vivo* multimodal imaging technologies, including PET, MRI, and fluorescence imaging to investigate the real-time events of tau-related neuropathology.

## MRI Studies for a Mouse Model of Tauopathy

Magnetic resonance imaging-based volumetry is a valuable tool for assessing disease progression in humans (15–19). As translational research of neurodegenerative diseases, several groups have reported studies using volumetric MRI in mouse models (20–25). Age-dependent volume reduction in both cerebral cortex and hippocampus of the rTg4510 mouse line was clearly demonstrated on MRI (24, 25). The volume (size) of the cerebral cortex from 5 to 8-month-old rTg4510 mice was significantly less (by approximately 20%) than that of age-matched non-transgenic (non-tg) mice (24). Brain atrophy in rTg4510 mice as examined by MRI was in agreement with previous histopathology findings (13, 26). A gender difference showing more severe phenotype in female rTg4510 mice compared with males (27) was confirmed by volumetric MRI study (24).

In addition to volumetric MRI, MR-based *in vivo* imaging techniques, including MR spectroscopy (MRS), manganese-enhanced MRI (MEMRI), arterial spin labeling, amide proton transfer imaging, and diffusion tensor imaging (DTI), have been tested to evaluate brain functions and microstructural changes in living rTg4510 mice (24, 25, 28–32). MEMRI is a technique for measuring neuronal function, because the manganese ion (Mn^2+^) accumulates in actively firing neurons through voltage-gated Ca^2+^ channels and serves as a positive contrast agent in T1-weighted brain images (33, 34). Using this technique, Perez et al. observed a reduction in both neuronal activity and volume in the hippocampus of 6-month-old rTg4510 mice (25). Fontaine et al. demonstrated neuronal dysfunction in the CA3 and CA1 regions of 3-month-old rTg4510 mice (32). Together, these studies illustrate the importance of the MEMRI method as a diagnostic research tool for the early detection of neuronal dysfunction in rTg4510 mice.

Diffusion tensor imaging is another MR-based imaging technique, which is applied to assess the integrity and organization of myelinated structures such as axons in white matter (WM) and also in gray matter regions (35). For this purpose, we used DTI to examine age-related alterations in WM diffusion anisotropy in rTg4510 and non-tg control mice (29). Our results indicated that 8-month-old rTg4510 mice show significant reductions in fractional anisotropy (FA) in WM structures compared with control non-tg and young (2.5-month-old) rTg4510 mice. The microstructural changes contributing to these age- and tauopathy-associated WM changes were supported by electron microscopic evidence of disorganized axonal processes and the presence of interprocess spaces, which could explain the reduced FA in aged rTg4510 mice (29). Reduced FA at a later age (over 7.5 months) was confirmed by another research group (30, 31). Since, reduced FA values in WM were mostly observed at an age that included the presence of significant atrophy and tau pathology, this standard method may be useful for assessing the therapeutic efficacy of treatments that may be used to reduce or prevent neurodegenerative progression. On the other hand, examination with additional diffusion anisotropy indices such as the mode of anisotropy allowed us to detect early signs (at 2.5 months old) of WM disorganization (29). Although disorganization of myelin morphology at this age was not determined, WM degeneration may be one of the early signs of tauopathy. Nevertheless, reduced WM integrity associated with tau pathology was confirmed by *in vivo* DTI studies.

## Tau PET Tracers

Current tau PET tracers are mostly designed by β-sheet binding properties. In principle, filamentous tau aggregates (e.g., NFTs, neuropile threads, tufted astrocytes, astrocytic plaques, and coiled bodies) will be labeled by these tracers with distinct specificity and selectivity. Up to date, three types of radiotracers have been widely tested for the clinical assessment of patients with tauopathy [reviewed in Ref. (36)]. These tracers include the arcyquinoline derivative THK5351 (37), the pyrido-indole derivative AV-1451 (38, 39), and the phenyl/pyridinyl-butadienyl-benzothiazole/benzothiazolium derivative PBB3 (4). Screening of potential tracers was performed by *in vitro* binding assays in AD brain homogenates and autoradiographies of AD brain sections. [^18^F]THK5351, [^18^F]AV-1451, and [^11^C]PBB3 showed good affinity for tau deposits and no selectivity of amyloid plaques. Accumulating evidence has shown that tau PET using these tracers might not detect pretangles in tauopathy brains (9, 40). Comparative *in vitro* binding assay of [^18^F]AV-1451 and [^11^C]PBB3 revealed that the binding of [^11^C]PBB3 to non-AD-type tau pathology (e.g., tufted astrocytes, astrocytic plaques, and pick bodies) was higher than that of [^18^F]AV-1451 (9). Another comparative study of [^3^H]AV-1451 and [^3^H]THK523 showed a distinct binding affinity for NFTs (41). Potential off-target bindings of [^18^F]AV-1451 and [^18^F]THK5351 to monoamine oxidase A (MAO-A) and MAO-B, respectively, were also observed (36, 42, 43). Although binding properties of tau PET tracers have been vigorously investigated for the past decade, the specificity and selectivity of PET signals to tau pathology are still to be fully understood.

## Tau PET Imaging Studies of rTg4510 Mice

Micro-PET imaging of tau pathology in tg mouse models of tauopathy has contributed to the characterization of novel PET tracers. Using [^18^F]THK523, micro-PET imaging in 6-month-old rTg4510 mice successfully showed significantly higher radiotracer retentions in brains compared with non-tg or PS1/APP mice (44). [^11^C]PBB3-PET imaging in another tauopathy mouse model, PS19 tg (expressing P301S mutant human tau) (4), and [^18^F]THK5117-PET imaging in P301S tg and biGT (bigenic GSK-3β × P301L tau) tg mice (45) were reported to show higher tracer uptake in tg than in non-tg mice. On the other hand, there was no significant difference in retention of [^18^F]AV-1451 between TAPP (bigenic APPswe × P301L tau) tg and non-tg mice (39). Inconsistency of these micro-PET analyses was mostly due to the use of different tg mouse models. Technical limitations also stem from the diversity of pathological characteristics in different mouse models. The distribution of tau deposits and the time course of pathological tau accumulation in tg mice differed from each other. It is very important to compare *in vivo* bindings of different tracers with reproducible mouse models. To initiate the development of a screening platform for tau PET tracers, our group has recently demonstrated longitudinal micro-PET imaging in rTg4510 mice (see text footnote 1). Consistent with neuropathological and biochemical observations, our [^11^C]PBB3 PET imaging of rTg4510 mice showed an age-dependent increase in [^11^C]PBB3 signal and that [^11^C]PBB3 retention was inversely correlated with neocortical volumes. The increasing [^11^C]PBB3 signal reached a plateau by 7 months of age. The correlation between [^11^C]PBB3 levels and brain atrophy disappeared in rTg4510 mice over 7 months old. Since, tau pathology is tightly linked to neuronal loss, the disappearance rate of [^11^C]PBB3-positive neurons may increase at age over 7 months. It should be noted that microglial activation in rTg4510 brains examined using translocator protein (TSPO) (18-kDa TSPO)-PET imaging showed significant correlation with both [^11^C]PBB3 level and brain atrophy (see text footnote 1). rTg4510 will be a useful model for investigating the mechanisms of tau-induced neuroinflammation. Nevertheless, in combination with tau PET imaging, the imaging of neuroinflammation will offer an additional diagnostic parameter for tauopathy.

## *In Vivo* Fluorescence Imaging for Detecting Tau Pathology

The invention of two-photon excitation laser scanning microscopy has given new impetus to the research field of investigating *in vivo* brain cell dynamics (46). In combination with newly developed fluorescence imaging techniques, cellular and molecular mechanisms underlying brain functions and impairments can be examined using *in vivo* animal models. In theory, the spatial scales of two-photon microscopy are from a micron to a millimeter. The side length of field-of-views can reach a few 100 μm. The imaging depth reached has been up to ~1 mm depending on the properties of the tissue (47). Using this technology, AD mouse models have been investigated with regard to monitoring the time course of disease progression [reviewed in Ref. (48)]. Hyman’s group first reported *in vivo* visualization of amyloid plaques in APP tg mouse lines (PDAPP mice and tg2576 mice) (49, 50). Clearance of plaques by anti-Aβ antibody was monitored by two-photon microscopy after Thioflavin S injection into the brain. Fluorescence imaging using Pittsburgh Compound B (PiB), which is used for amyloid PET imaging, was also examined to visualize amyloid plaques in APP tg mouse lines (51). As a result, the multimodality of PiB ligand for both fluorescence and PET imaging was clearly demonstrated. Because the PBB3 ligand was derived from the same tracer family as the PiB ligand, a similar approach was used to visualize the tau pathology in PS19 tg mice (4). Moreover, the current chronic cranial window setting enables the use of long-period two-photon imaging for more than 2 months (52). As for our study, longitudinal monitoring of PBB3-positive neurons has been performed in living rTg4510 mice (Takuwa et al., manuscript in preparation). Our data showed that PBB3-positive inclusions were visualized with two-photon imaging at an age of as early as 4 months, with fluorescence signals then reaching a plateau at 6 months. These data are in agreement with both [^11^C]PBB3 PET imaging and volumetric MRI, suggesting multimodal imaging utilities of the PBB3 ligand. On the other hand, Hyman’s group has investigated the mechanism of neuronal loss in rTg4510 mouse brains using the fluorescence indicator of caspase activation and β-sheet ligand X-34 (a Congo red derivative) (53). Their data indicate that NFT formation has a neuroprotective effect against caspase-mediated neuronal death. Synaptic dysfunction is another key event taking place during the pathogenesis of tauopathy. Previous reports showed decreased dendritic spine and synapse density in cortical slices prepared from 9 to 10-month-old rTg4510 mice (54, 55). Most recently, Jackson et al. demonstrated the visualization of synapses in the somatosensory cortex of rTg4510 mice by two-photon microscopy after the injection of adeno-associated virus that drove neuronal expression of either GFP or GCaMP6m (56). For longitudinal imaging, GFP-expressing axonal and dendritic regions were imaged weekly to investigate the turnover of axonal terminal boutons and dendritic spines. For functional imaging, GCaMP6-expressing neurons were imaged in lightly anesthetized animals to measure neuronal activity in response to whisker stimulation. The authors observed mismatched abnormalities in pre-and post-synaptic turnover coinciding with disrupted neuronal activity at 5 months of age. Their data suggests that synaptic dysfunction precedes tangle-associated neurodegeneration. Although linkage between aggregated tau formation and synaptic dysfunction remains unclear, *in vivo* monitoring of tau pathology at cellular levels provides an advantage for dissecting the mechanisms of tau toxicity.

## Design for a Diagnostic Platform of Tauopathy

**Figure 1:**
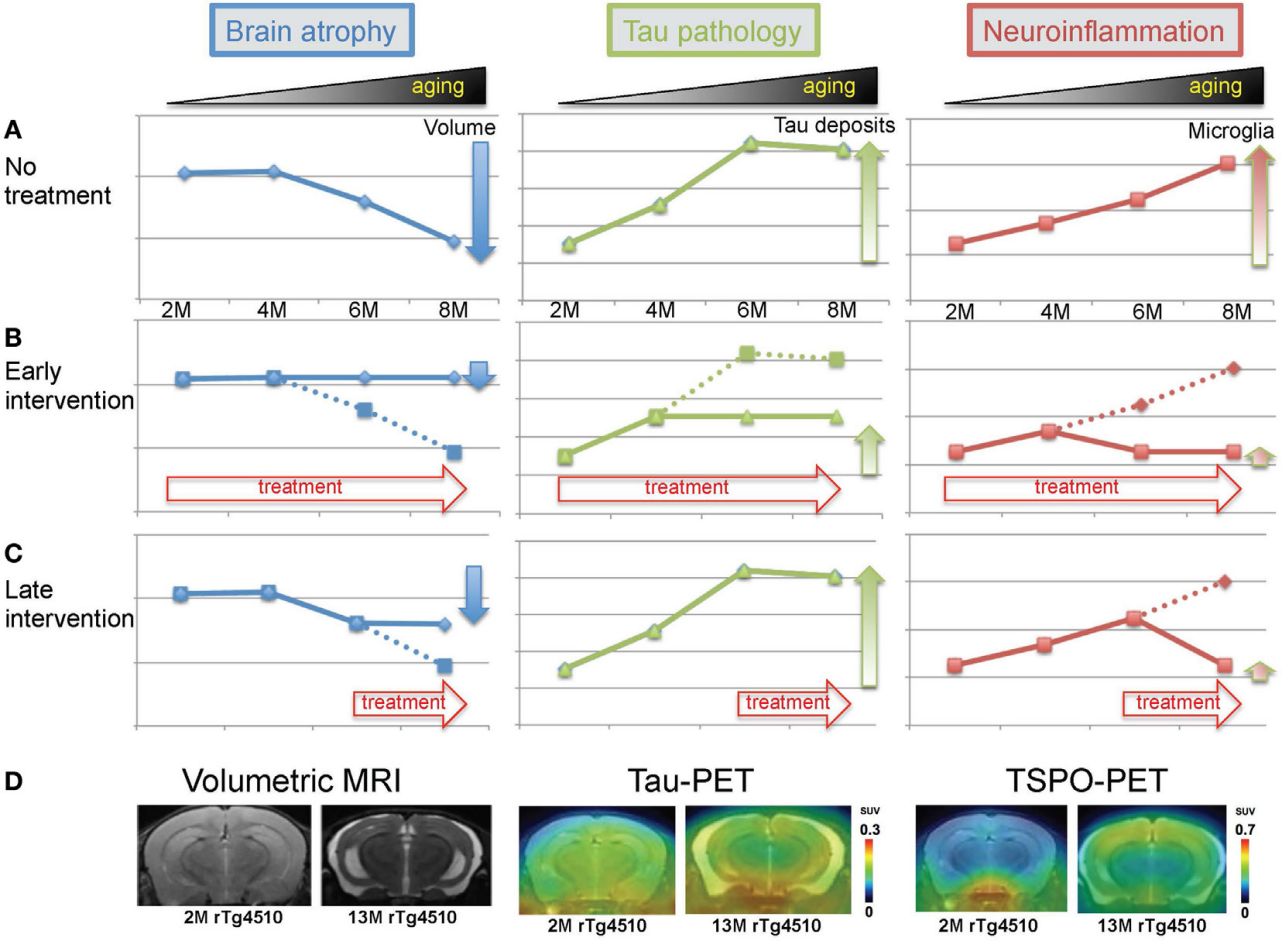
Imaging modalities for investigating tau-induced brain atrophy and neuroinflammation. **(A)** Theoretical processes of volume change, pathological tau accumulation, and microglial activation in rTg4510 mice. **(B)** Effects of early intervention. Treatment was started from 2 months of age. **(C)** Effects of late intervention. Treatment was started from 6 months of age. **(D)** Representative images of volumetric magnetic resonance imaging (MRI), tau-positron emission tomography (PET), and translocator protein-PET in 2- and 13-month-old rTg4510 mice.

The rTg4510 mouse is one of the widely used models of tauopathy. As described above, several groups have conducted *in vivo* imaging studies on rTg4510 mice (Table 1). Because the time course of tau pathology and forebrain atrophy have been well examined using postmortem materials, experimental designs for drug intervention can be easily designed (Figure 1). Since, this mouse model allows the tetracycline-repressible overexpression of human tau, doxycycline treatment for the suppression of tau expression will provide a positive control in an experimental design of the evaluation of therapeutic candidates. Previous study showed that suppression of tau from 5.5 months of age reversed memory deficits, while tangles persisted and continued to accumulate (11). Therefore, early intervention starting at 2 months of age could be more effective to prevent both NFT formation and brain atrophy (Figure 1B). Although effects will be limited, late intervention would be worthwhile if aggravation of tau-induced brain atrophy can be slowed down (Figure 1C). In our study, we developed a unique diagnostic platform of *in vivo* imaging of volumetric MRI, tau-PET, and TSPO-PET (Figure 1D). Brains of living rTg4510 mice can be used to monitor their volume, pathological tau accumulation, and neuroinflammation. Moreover, live cell imaging with two-photon microscopy allows us to capture NFT formation and neuronal death in rTg4510 mice. Using these platforms, we expect to be able to validate several drug candidates in the foreseeable future.

**Table 1:** Reference list for *in vivo* imaging in living rTg4510 mice.

Imaging system	Imaging techniques	Reagents and application for *in vivo* imaging	Reference
Magnetic resonance imaging (MRI)	Volumetric MRI ^1^H MR spectroscopy		Yang et al. (24) Neuroimage
MRI	Manganese enhanced MRI (MEMRI)	Manganese (intraperitoneal injection)	Perez et al. (25) Mol. Neurodegeneration
MRI	MEMRI	Manganese (nasal lavage)	Majid et al. (28) Neuroimage Clin.
MRI	Diffusion tensor imaging (DTI)		Sahara et al. (29) Neurobiol. Aging
MRI	Volumetric MRI, DTI Arterial spin labeling (ASL), Exchange saturation transfer (CEST), glucose CEST	Glucose (intraperitoneal injection)	Wells et al. (30) Neuroimage
MRI	Volumetric MRI, DTI, ASL, CEST		Holmes et al. (31) Neurobiol. Aging
MRI	MEMRI	Manganese (intraperitoneal injection)	Fontaine et al. (32) Neurobiol. Aging
PET	PET	r^18^FlTHK523	Fodero-Tavoletti et al. (44) Brain
PET MRI	PET volumetric MRI	r^11^ClPBB3 ^11^CIAC-5216 (TSPO)	(see text footnote 1)
Two-photon microscopy	Fluorescence imaging	Thioflavin S (intracerebral injection)	Spires-Jones et al. (57) J Neurosci.
Two-photon microscopy	Fluorescence imaging	Thioflavin S (intracerebral injection) X-34 (i.v.)	De Calignon et al. (53) Nature
Two-photon microscopy	Fluorescence imaging	Thioflavin S (intracerebral injection)	Kopeikina et al. (58) PLoS One
Two-photon microscopy	Fluorescence imaging	Thioflavin S (intracerebral injection)	Kuchibhotla et al. (59) PNAS
Two-photon microscopy	Fluorescence imaging	GFP (AAV serotype2) GCaMP6m (AAV 1/2 hybrid serotype)	Jackson et al. (56) Cell Rep.

## Conclusion

For more effective therapies, the pre-clinical evaluation of drugs for tauopathy is needed. The use of animal models that recapitulate the critical features of the disease, such as NFTs, cognitive impairment, brain atrophy, and neuronal loss, is essential. The rTg4510 mouse model of tauopathy fulfills the required features, despite the fact that tau protein was expressed at a non-physiologically higher level over the total life span. *In vivo*, brain imaging offers reliable approaches to validating the pathological status and to determining the efficacy of drugs for exploring disease-modifying therapies.

## Author Contributions

Authors wrote and proofed the manuscript.

## Conflict of Interest Statement

The authors declare that the research was conducted in the absence of any commercial or financial relationships that could be construed as a potential conflict of interest.

## Footnotes

^1^Ishikawa I, Tokunaga M, Maeda J, Minamihisamatsu T, Shimojo M, Takuwa H, et al. In vivo visualization of tau accumulation, microglial activation and brain atrophy in a mouse model of tauopathy rTg4510. *J Alzheimers Dis* (2018, Forthcoming).
